# Improving the efficiency of using multivalued logic tools: application of algebraic rings

**DOI:** 10.1038/s41598-023-49593-1

**Published:** 2023-12-12

**Authors:** Ibragim E. Suleimenov, Yelizaveta S. Vitulyova, Sherniyaz B. Kabdushev, Akhat S. Bakirov

**Affiliations:** 1 National Engineering Academy of the Republic of Kazakhstan, Bogenbai Batyr Str. 80, 050010 Almaty, Kazakhstan; 2 Gumarbek Daukeyev Almaty University of Power Engineering and Telecommunications, Baytursynov Str. 126/1, 050013 Almaty, Republic of Kazakhstan; 3 Al-Farabi Kazakh National University, Almaty, 050040 Republic of Kazakhstan

**Keywords:** Applied mathematics, Computer science, Information technology

## Abstract

It is shown that in order to increase the efficiency of using methods of abstract algebra in modern information technologies, it is important to establish an explicit connection between operations corresponding to various varieties of multivalued logics and algebraic operations. For multivalued logics, the number of variables in which is equal to a prime number, such a connection is naturally established through explicit algebraic expressions in Galois fields. It is possible to define an algebraic δ-function, which allows you to reduce any truth table to an algebraic expression, for the case when the number of values accepted by a multivalued logic variable is equal to an integer power of a prime number. In this paper, we show that the algebraic δ-function can also be defined for the case when the number of values taken by a multivalued logic variable is p − 1, where p is a prime number. This function also allows to reduce logical operations to algebraic expressions. Specific examples of the constructiveness of the proposed approach are presented, as well as electronic circuits that experimentally prove its adequacy.

## Introduction

Multivalued logic has been developing quite actively in recent decades^1,2^. Various varieties of multivalued logics have been developed, in particular, paralogics^3^, including paracomplete^4^ and paraconsistent^5^ logic. Closely related to them, multivalued algebraic structures of various kinds also attract the attention of researchers^6,7^. Works in the field of multivalued logic are also aimed at practical application, in particular, various kinds of computational and expert systems using multivalued logic are being developed^8,9^. There are reports in the literature in which multivalued logic is used to improve neural networks^10,11^.

The application of multivalued logic to the construction of neural networks obviously also has a pronounced philosophical aspect^12,13^. Human thinking obviously cannot be reduced to binary logic it operates with such categories as modality, which obviously do not correspond to concepts built on the opposition "True–False". It is appropriate to emphasize that it was precisely the attempts to go beyond this opposition that led to the appearance of the pioneering works of Lukasevich^14^ and Vasiliev^15^, which gave rise to research in the field of multivalued logic. Moreover, as shown in^13^, a fundamental feature of human thinking is the ability to lie, which is inseparable from any form of creativity. At a minimum, the human mind can operate with contradictions as well, which determines the increased interest in paracomplete^4^ and paraconsistent^5^ logics.

Philosophical understanding of the essence of intelligence is becoming more and more in demand due to the need to improve artificial intelligence (AI) systems^16,17^. As emphasized in^18^, discussions about whether this system can be considered as artificial intelligence or not are pointless, since the essence of intelligence as such remains undisclosed.

The question of what kind of “logic” the human intellect operates on remains open. Moreover, there is every reason to believe that he can "switch" from one "logic" to another.

This makes even more relevant the question of how logics of various types are related to each other, as well as the question of whether one or another of them can be reduced to others. This issue is far from being completely resolved^19^, but there is no doubt that the reduction of multivalued logics to algebraic form^20^ can become an important tool for solving it.

Reducing the operations of multivalued logic to an algebraic form is also of interest from the point of view of improving fuzzy logic^21–23^, since the variables of multivalued logic can be put in correspondence with the variables of fuzzy logic. This issue is also of interest from the point of view of using multivalued logic to improve neural networks^24,25^. Non-binary logics are of interest from the point of view of bringing algorithms for the functioning of neural networks to an explicit form^26,27^.

Reducing the operations of multivalued logic to algebraic form is most easily done when it is possible to establish a one-to-one correspondence between the values of the variables of multivalued logic and the elements of some Galois field. This is possible only for ${p}^{n}$-logics, where $p$ is a prime number, $n$ is an integer. As follows from the theory of Galois fields^28^, such fields can contain only ${p}^{n}$ elements, including zero.

We emphasize that the following result, obtained within the framework of classical mathematical logic operating with binary variables, is fundamental for modern computer technology. Any binary operation can be reduced to two others. This allows us to reduce all binary logic operations to addition and multiplication operations in the Galois field $GF(2)$. The algebraic δ-function defined in^18^ makes clear the previously made conclusion that if the number of values that a logical variable can take is equal to ${p}^{n}$, where $n$ is an integer and $p$ is a prime number, then all operations of such logic can also be reduced to the operations of addition and multiplication over the Galois field $GF({p}^{n})$.

The question arises as to what kind of algebraic operations the operations of multivalued logics that do not satisfy the above criterion can be reduced to.

Examples of such logics are known they include six-valued ones^29^, as well as ten-valued logic, which is of direct practical interest, since the decimal number system remains the most common.

In this paper, we show that the approach proposed in^20^ can be generalized to the case when the number of values of multivalued logic variables is equal to $p-1$.

Specifically, an algebraic δ-function for this case can also be introduced into consideration. Examples of such logics discussed in this work are six- and ten-valued logic.

The tool for generating the algebraic δ-function for the cases under consideration is the transition from the use of algebraic fields to finite algebraic rings, which are already widely used in information technology^30,31^.

It is significant that the operations of the above logics turn out to be irreducible to the operations of addition and multiplication in conjugate algebraic structures. In relation to logics complementary to Galois fields $GF\left(p-1\right)$, it is required to use the digital logarithm operation, as well as its inverse.

Thus, we show that there are examples of logics whose algebraization requires the use of a larger number of operations than is the case in classical cases.

Further, along with the reduction of multivalued logic operations to an algebraic form, it is also of interest to develop devices, including electronic devices that implement such or similar operations^32,33^. They are also of direct practical interest, for example, for the development of measuring equipment^34^.

Electronic circuits that implement basic operations for the six-valued logic case, which is important, are also presented in this paper. Among other things, this also makes it possible to verify the conclusions and calculations made using simulation tools.

We also note that the issues under consideration are also important from the point of view of improving the methods of digital signal and image processing. As shown in^30,35,36^, it is permissible to use functions that take values in Galois fields and/or algebraic rings to simulate digital signals. This allows you to move on to signal processing tools based on multivalued logic.

Section "Reduction of operations of -valued logic to algebraic form" shows that there is an explicit expression that converts $p-1$-valued logic operations to algebraic form.

Section "Visual demonstration of the connection between algebraic rings and algebraic fields" shows a visual connection between Galois fields containing p elements and algebraic rings containing $p-1$ elements.

Section "Digital logarithm operation for the field and its circuit implementation" discusses the digital logarithm operation, which establishes a connection between Galois fields containing $p$ elements and algebraic rings containing $p-1$ elements, in relation to an important special case of six-valued logic. An electronic circuit is presented that ensures the performance of the corresponding operations. It is proved that six-valued logic can be reduced to (2,3)-logic by using the theory of algebraic rings.

Section "Modulo 6 multiplication algorithm and multiplier circuit" discusses specific electronic circuits that prove the constructiveness of the proposed approach, in particular, circuits that perform the multiplication operation for (2,3) logic, to which six-valued logic is reduced.

## Reduction of operations of ${\varvec{p}}-1$-valued logic to algebraic form

One of very interesting (and far from being completely solved) problems, important for further development of information technologies based on multivalued logic, is the development of digital logarithm methods^37,38^. The operation of digital logarithm allows to reduce the operation of multiplication of two Galois field elements to the operation of addition, and the operation of magnification to the operation of multiplication.

As noted in the “Introduction”, for multi-valued logics, complementary Galois fields $GF({p}^{n})$ (i.e. for the case when the number of values that a logical variable can take is equal to ${p}^{n}$, where $p$ is a prime number, $n$ is an integer), any logical operations can be reduced to addition and multiplication operations in a complementary Galois field. For the case when this condition is not met, it is necessary to expand the list of operations used to reduce logical operations to algebraic ones. In the case under consideration, such an additional operation is the operation of digital logarithm, as well as its inverse.

Recall that any element of the Galois field $GF(p)$ satisfies the equality

1 $${x}^{p-1}=1.$$

Each nonzero element of the field can be represented as

2 $$x={\theta }^{n}, n=0, 1,\dots , p-1,$$

where $\theta$-primitive element.

By virtue of relations (1) and (2) we have

3 $${\theta }^{n}{\theta }^{m}={\theta }^{n+m, mod(p-1)}.$$

Ratio (3) shows that the operation of multiplication modulo p, can be reduced to the operation of addition modulo $p-1$. This, however, requires a numerical logarithm operation, i.e., finding an algorithm that allows one to set the number $n$ to a given $x$.

This problem can be solved, for example, starting from the analogue of the Zhegalkin polynomial given in^20^, which can be constructed as follows. The following expression may be treated as a logical analogue of the δ-function^20^.

4 $${\delta }_{i}\left(x\right)=1-{\left(x-{x}_{i}\right)}^{p-1},$$

where ${x}_{i}$ is a fixed element of the field $GF(p)$.

Indeed, due to expression (1), functions ${\delta }_{i}\left(x\right)$ have the following property

5 $${\delta }_{i}\left(x\right)=\left\{\begin{array}{c}1, x={x}_{i}\\ 0, x\ne {x}_{i}\end{array}\right..$$

Let us consider next polynomial

6 $$F\left(x,y\right)=\sum_{i,j=0}^{i,j=p-1}f\left({x}_{i},{y}_{j}\right){\delta }_{i}\left(x\right){\delta }_{j}\left(y\right),$$

where the values $f({x}_{i},{y}_{j})$ form a truth table.

When a particular pair of ${x}_{{i}_{0}},{y}_{{j}_{0}}$ elements corresponding Galois field is substituted into expression (6), all summands appearing in the sum in the right part of formula (6) turn to zero except the summand for which $i={i}_{0},j={j}_{0}$ is satisfied. Hence,

7 $$F\left({x}_{{i}_{0}},{y}_{{j}_{0}}\right)=f\left({x}_{{i}_{0}},{y}_{{j}_{0}}\right).$$

Expression (6), among other things, makes it possible to reduce any binary operation of multivalued logic to an explicit algebraic expression when the number of variables of this logic is equal to a prime number, i.e., the set of logic variables can be put into a one-to-one correspondence with the set of elements of a certain Galois field.

Similarly, an expression can be constructed that provides the digital logarithm operation.

Indeed, expression (6) is built based on a table reflecting a specific binary operation of multivalued logic. A similar table can be constructed explicitly for the digital logarithm operation. Indeed, for each specific $\theta$ one can specify a specific integer n that corresponds to a given element of the field $x$.

Formally, we can write

8 $$dl\left(x\right)=\sum_{i=1}^{i=p-1}dl\left({x}_{i}\right){\delta }_{i}\left(x\right),$$

where $dl\left(x\right)$ denotes the digital logarithm of the field element $x$.

However, notation (8) makes sense only insofar as the value $dl\left({x}_{i}\right)$ is an element of the Galois field used. This means that this expression is useful only when there is an easy way to specify another correspondence between the integers n and the elements of the Galois field being used.

Obviously, this is easiest to do when the fields $GF(p)$ are used. In this case, each element of the field can be given a corresponding integer or zero.

We also emphasize that in formula (8) the lower summation limit is changed to 1, which corresponds to the fact that the digital logarithm of zero does not make sense. Six non-zero elements of the field $GF(7)$ are associated with six elements of the same field. This set includes 0 but does not include the element corresponding to the number 6.

It is easy to pass from expression (8) to an expression that is applicable for the algebraic description of multivalued logic operations, the number of variables in which is equal to $p-1$.

9 $$Q\left(x,y\right)=\sum_{i,j=0}^{i,j=p-2}Q\left({x}_{i},{y}_{j}\right){\delta }_{i}\left({\theta }^{n\left(x\right)}\right){\delta }_{j}\left({\theta }^{n\left(y\right)}\right).$$

In this expression, the "algebraic δ-function"

10 $${\delta }_{i}\left(x\right)=1-{\left({\theta }^{n\left(x\right)}-{\theta }^{n\left({x}_{i}\right)}\right)}^{p-1},$$

applied to the power of the primitive element $\theta$, which uses the correspondence between multivalued logic variables and integers.

In the written expressions, in fact, a well-defined mapping of the field $GF(p)$ onto an algebraic ring, which contains $p-1$ elements, is actually used. Let's consider this connection in more detail. This, among other things, is usefull for the construction of electronic circuits that perform the digital logarithm operation.

## Visual demonstration of the connection between algebraic rings and algebraic fields

The number p is prime, but the number $p-1$ is no longer prime. The only exception is the case of $p=2$, which is of no practical interest.

Hence, there are zero divisors in the ring of modulo $p-1$ deduction classes, and their existence is a clear sign that distinguishes rings from fields. Otherwise, calculations using the transition from an element $x={\theta }^{n}$ to $n$, actually corresponds to transition from calculations in terms of an algebraic field to calculations in terms of an algebraic ring.

The functioning algorithm of the considered electronic circuits, discussed below and used to illustrate the proposed approach, is based on the well-known theorem from the theory of algebraic rings, according to which there are rings $R$, decomposing into a direct sum of ideals ${r}_{i}$

11 $$R={r}_{1}+{r}_{2}+\dots +{r}_{n}.$$

Each of these ideals is generated by idempotent elements ${e}_{i}$

12 $${r}_{i}=R{e}_{i},$$

which cancel each other out

13 $${e}_{i}{e}_{j}=0, i\ne j; {e}_{i}{e}_{i}={e}_{i},$$

and their sum is equal to one of the ring under consideration

14 $$\sum_{i}{e}_{i}=1.$$

An example of such a ring is a ring obtained through a homomorphic mapping of a ring of integers to a ring of classes of deductions modulo 6 (this ring can be assigned to a set of six-valued logic variables). In this case any positive integer less than 6 can be represented as

15 $$u=3\cdot {u}_{1}+4\cdot {u}_{2},$$

where ${u}_{\mathrm{1,2}}$ take the following values

16 $${u}_{1}=\mathrm{0,1}; {u}_{2}=\mathrm{0,1},2.$$

It can be seen that in operations modulo 6 the elements of the ring of deduction classes appearing in (15) really act as idempotent elements, i.e., the following takes place

17 $$3\cdot 3=9\equiv 3\left(6\right),4\cdot 4=16\equiv 4\left(6\right).$$

Moreover, these elements cancel each other out,

18 $$3\cdot 4=12\equiv 0\left(6\right),$$

and their sum modulo 6 is one

19 $$4+3=7\equiv 1\left(6\right).$$

One can see that the ring of deduction classes under consideration is indeed an example of the fulfillment of relations (11)–(14). Moreover, this example emphasizes that the number of elements in the ideals into which the ring splits does not necessarily have to be the same.

The number 6 is the product of prime numbers 3 and 2, so the ideals generated by the idempotent elements correspond to Galois fields $GF(3)$ and $GF(2)$. They contain 3 and 2 elements, respectively, as relations (16) show.

Accordingly, formula (15) can be viewed as a representation of a number in binary ternary logic. In particular, instead of the notation (15) one can use its abbreviated version

20 $$u={u}_{1}{u}_{2},$$

where numbers ${u}_{1}{u}_{2}$ и ${u}_{1}{u}_{2}$ are treated as analogues of decimal places (the analogy with the writing of decimal numbers is obvious).

Note also that formula (8) also allows us to represent all non-zero elements of Galois field $GF(7)$ in the following form

21 $$x={\theta }^{3\cdot {u}_{1}}{\theta }^{4\cdot {u}_{2}}={g}_{1}^{{u}_{1}}{g}_{2}^{{u}_{2}}.$$

where elements ${g}_{i}$ are determined from the conditions

22 $${g}_{1}^{2}={g}_{2}^{3}=1.$$

Elements ${g}_{i}$ for the case in question can be chosen as follows

23 $${g}_{1}=6; {g}_{2}=2,$$

which is proved by direct verification.

This choice is not the only one, in particular, one can put ${g}_{2}=4$.

The advantage of representation (15) is that the digit analogs can be handled independently. Indeed, consider the product modulo 6 of two numbers written in the form (15)

24 $$u={e}_{1}{u}_{1}+{e}_{2}{u}_{2}.$$

Considering that ${e}_{\mathrm{1,2}}$ are idempotent elements and that the sets of variable values ${u}_{\mathrm{1,2}}$ are isomorphic to the Galois fields generated by prime numbers ${p}_{\mathrm{1,2}}$, we have

25 $${u}^{\left(1\right)}{u}^{\left(2\right)}={e}_{1}{u}_{1}^{\left(1\right)}{u}_{1}^{\left(2\right)}+{e}_{2}{u}_{2}^{\left(1\right)}{u}_{2}^{\left(2\right)}.$$

The same result is true for the addition operation.

26 $${u}^{\left(1\right)}+{u}^{\left(2\right)}={e}_{1}\left[{u}_{1}^{\left(1\right)}+{u}_{1}^{\left(2\right)}\right]+{e}_{2}\left[{u}_{2}^{\left(1\right)}+{u}_{2}^{\left(2\right)}\right],$$

where the addition in square brackets is made by the modulus of the number specifying the corresponding digit analog.

Further, the formula (21) makes it possible to demonstrate the specificity of the search for primitive elements. In particular, it immediately follows that the primitive element in the choice of elements ${g}_{i}$ according to formula (23) is

27 $$\theta ={g}_{1}{g}_{2}=5.$$

Degrees of this element form gives all non-zero elements of field $GF\left(7\right)$.

Another important example involves converting ten-valued logic operations to algebraic form. This field in the sense of the algebraic delta function (10) is conjugate to the field $GF(11)$, i.e. operations in an algebraic ring corresponding to ten-valued logic are reduced to algebraic ones through the use of addition and multiplication operations in the $GF(11)$ field, as well as the operation of digital differentiation.

In this case, any positive integer less than 10 can be represented as

28 $$u=5\cdot {u}_{1}+6\cdot {u}_{2},$$

where ${u}_{\mathrm{1,2}}$ take the following values

29 $${u}_{1}=\mathrm{0,1};{u}_{2}=\mathrm{0,1},\dots ,4.$$

It is easy to see that in this case analogues of formulas (17)–(19) are also satisfied, in particular

30 $$5+6=11\equiv 1\left(10\right).$$

Accordingly, the field element $GF(11)$ included in the expression for the algebraic delta function (10) can be represented in a form similar to (21)

31 $$x={\theta }^{5\cdot {u}_{1}}{\theta }^{6\cdot {u}_{2}}={g}_{1}^{{u}_{1}}{g}_{2}^{{u}_{2}}.$$

where elements ${g}_{i}$ of the $GF(11)$ field, are determined from next conditions

32 $${g}_{1}^{2}={g}_{2}^{3}=1.$$

In particular, we can choose ${g}_{1}=10; {g}_{2}=3$.

Note that the case of 10-valued logic may be of interest, including from an applied point of view. Namely, computing systems based on non-trivial elemental base^39^, including quasi-biological^40^, are currently being actively developed. Since in the foreseeable future humanity is unlikely to abandon the decimal number system, bringing the operations of such logic to algebraic ones can potentially be used to create computing systems directly oriented towards the decimal number system.

Formulas (25) and (26) allow us to propose the algorithm of multiplication modulo 6 and the circuit of multiplier modulo 6 that implements it (a similar approach can be used for decimal logic). Its advantage is the ability to operate with the "digits" of the number represented by formula (20) independently. This algorithm is considered in section "Modulo 6 multiplication algorithm and multiplier circuit". However, it should be emphasized once again that the operations of addition and multiplication of ring elements do not provide the possibility of reducing all operations of $\left(p-1\right)$-logic to algebraic ones, as is the case in the fields $GF(p)$. These operations must be supplemented by the operation of digital logarithm and its inverse—exponentiation.

## Digital logarithm operation for the ${\varvec{G}}{\varvec{F}}(7)$ field and its circuit implementation

The importance of the digital logarithm operation for the case of $\left(p-1\right)$-logic follows directly from formula (10). Indeed, the algebraic delta function, which, as follows from formula (9), makes it possible to reduce any operations of such logic to algebraic ones, takes values in the field $GF(p)$. In fact, formula (10) operates with a mapping of an algebraic ring containing $\left(p-1\right)$ onto the field $GF(p)$. Therefore, to return to the original ring, i.e., to really ensure the execution of operations in terms of $p-1$-logic, it is necessary to have a tool that will provide the reverse transition. This is the digital logarithm operation.

Let us consider how exactly we can make the transition from $x={\theta }^{n}$ to $n$, i.e., perform the operation of digital logarithm for the field $GF(7)$. More precisely, we will show that, using relation (8), it is possible not only to provide such a transformation, but also to propose an electronic circuit that performs the digital logarithm operation.

For this purpose, we first transform relation (10). The next relation is proven in theory of algebraic fields

33 $${\left(y\pm x\right)}^{q}={y}^{q}\pm {x}^{q},$$

where $q$ is the characteristic of the field.

For fields $GF(p)$ the number $p$ coincides with the characteristic.

Direct verification proves the validity of the equality

34 $${y}^{p}-{x}^{p}=\left(y-x\right)\left({y}^{p-1}+{y}^{p-2}x+\dots +y{x}^{p-2}+{x}^{p-1}\right).$$

Substituting the ratio (33) in the right part of formula (34), we get

35 $${\left(y-x\right)}^{p-1}={y}^{p-1}+{y}^{p-2}x+\dots +y{x}^{p-2}+{x}^{p-1}.$$

For the special case of the field $GF(7)$, this relation becomes

36 $${\left(y-x\right)}^{6}=1+{y}^{5}x+\dots +y{x}^{5}+1.$$

We will use representation (21) for the elements of the field under consideration. Then, after simple transformations, expression (36) can be reduced to the form

37 $${\left(y-x\right)}^{6}-1=\left({g}_{1}^{n}{g}_{1}^{m}+1\right)\left({g}_{2}^{2n}{g}_{2}^{m}+{g}_{2}^{n}{g}_{2}^{2m}+1\right),$$

where $x={g}_{1}^{n}{g}_{2}^{n}$; $y={g}_{1}^{m}{g}_{2}^{m}$ and the relation (22) was used.

Substituting specific values, we get

38 $$-{\delta \left(x,y\right)=\left(y-x\right)}^{6}-1=\left({2}^{2n}{2}^{m}+{2}^{n}{2}^{2m}+1\right)\left({6}^{n}{6}^{m}+1\right).$$

It can be seen that for the case under consideration, the algebraic δ-function is factorized, since we have

39 $${2}^{2n}{2}^{m}+{2}^{n}{2}^{2m}+1=\left\{\begin{array}{c}3,m+n\equiv 0(3)\\ 0,m+n\not\equiv 0(3)\end{array}\right.,$$

40 $${6}^{n}{6}^{m}+1=\left\{\begin{array}{c}2,m+n\equiv 0(2)\\ 0,m+n\not\equiv 0(2)\end{array}\right..$$

In the case when the right side of expression (38) is not equal to 0, it is equal to 6, which in the field $GF(7)$ corresponds to the inverse (by addition) elements with respect to 1.

Expressions (39) and (40) can be put in accordance with the operations carried out in the binary representation of the elements of the field under consideration.

Indeed, the prime number 7 is a special case of Mersenne prime numbers represented as ${2}^{q}-1$. Such numbers have the following property. When you multiply a number written in binary form by 2 (modulo equal to a Mersenne number), there is a cyclic permutation of characters. In particular,

41 $$2\cdot {a}_{2}{a}_{1}{a}_{0}{=}_{\left(7\right)}{a}_{1}{a}_{0}{a}_{2},$$

where ${a}_{i}$—binary characters.

The binary digits of all numbers ${N}_{0}$ corresponding to non-zero elements of the field $GF(7$ are presented in Table 1. It also presents the degrees of $n$ primitive elements $\theta =5$ and $\theta =3$, which correspond to these elements.

**Table 1:** Binary representation of non-zero elements of the field $GF(7$ and their corresponding degrees of primitive elements.

${N}_{0}$	1	2	4	3	6	5
${a}_{2}$	0	0	1	0	1	1
${a}_{1}$	0	1	0	1	1	0
${a}_{0}$	1	0	0	1	0	1
$n; \theta =5$	$6\equiv 0(6)$	4	2	5	3	1
$n; \theta =3$	$6\equiv 0(6)$	2	4	1	3	5

This table emphasizes that the nonzero elements of the field $GF(7)$ decompose into two subsets corresponding to formulas (39) and (40). In each of them, the sequences of binary symbols corresponding to the field elements relate to each other by cyclic permutation (42).

Element 6, for which connection $1+6\equiv 0(3)$ is valid, provides a transition from element to the element inverse by addition. Namely,

42 $$6\cdot {a}_{2}{a}_{1}{a}_{0}{=}_{\left(7\right)}{\overline{a} }_{2}{\overline{a} }_{1}{\overline{a} }_{0},$$

where the slash above the symbol corresponds to the logical operation of inversion (logical 0 turns into one and vice versa).

The fact that the element ${\overline{a} }_{2}{\overline{a} }_{1}{\overline{a} }_{0}$ is the inverse of ${a}_{2}{a}_{1}{a}_{0}$ is also proved directly

43 $${a}_{2}{a}_{1}{a}_{0}+{\overline{a} }_{2}{\overline{a} }_{1}{\overline{a} }_{0}=111{=}_{(7)}000.$$

**Figure 1:**
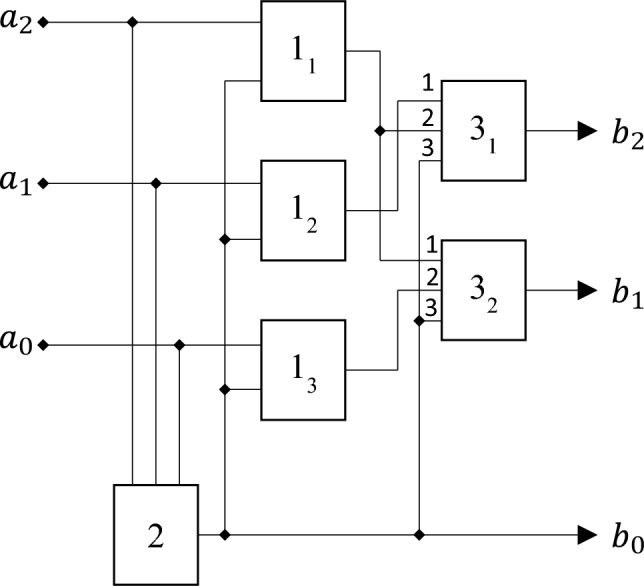
Circuit, performing the operation of digital logarithm.

The specificity of the field $GF(7$), expressed in Table 1 and formula (22), allows to realize the following circuit, performing the operation of digital logarithm (Fig. 1).

At the input of the circuit the binary signals ${a}_{i}$, corresponding to the elements of the field $GF(7$), Table 1, are received. The purpose of this circuit is to convert such signals into a set of binary signals ${b}_{i}$, corresponding to the exponent of degree $n$ at a certain choice of a primitive element. Concretely, the considered scheme corresponds to the case $\theta =5$.

Elements 1_1–3_ and element 2 provide the transformation of the set of input signals into the format when only one of them is non-zero. This corresponds to the use of the second factor on the right side of formula (38). As follows from Table 1, the conversion to such format is performed by inversion of all signals ${a}_{i}$, which is performed if the sum of $\sum {a}_{i}$ exceeds one.

Specifically, three elements 1, 2, and 4 from the original field (Table 1) differ from elements 3, 5, and 6 in that for the elements of the first of these groups, the sum of the values of all three digits is equal to 1, and for the second, it is equal to 2. At the same time, as follows from formula (42), there is a one-to-one relationship between the elements of each of these groups, determined by logical inversion, in which the value of the digit 0 goes to 1 and vice versa.

Therefore, it is possible to determine by simple means which of the groups a particular element belongs to, displayed by a sequence of binary characters ${a}_{2}{a}_{1}{a}_{0}$, which correspond to the values at the inputs of the circuit, Fig. 1.

This operation is performed by block 2, Fig. 1, which performs the summation of the values of the bits $\sum {a}_{i}$.

At the output of this block is formed the logical one if $\sum {a}_{i}>1$ and logical zero otherwise.

Further, the signals ${a}_{i}$ are fed to the EXCLUSIVE OR elements 1_1–3_, to the second inputs of which the signal from element 2 is fed. As a result, elements 1_1–3_ carry out a logical inversion of each of the signals arriving at the inputs of the circuit in Fig. 1 if $\sum {a}_{i}>1$ and leave them unchanged otherwise.

Further operations, performed by the circuit of Fig. 1, correspond to Table 2. In this table, the elements of the $GF(7)$ field are grouped in the same way as in Table 1. The difference is that in Table 2 shows not the values of the binary digits themselves, corresponding to the signals coming to the input of the circuit Fig. 1, but the values of the bits ${\widetilde{a}}_{j}$, formed at the outputs of elements 1_1–3_, i.e. the values for elements 1, 2 and 4 remain unchanged, and the values of the bits for elements 3, 6 and 5 are changed to logically inverted. This is emphasized by the first line of Table 1, which indicates the algebraic operation (multiplication by the element 6 in the Galois field $GF(7)$), which provides the inversion.

**Table 2:** Table of logical conversion of digit values at digital logarithm in the field $GF(7$).

	No inversion			Inversion		
	1	2	4	6*3	6*6	6*5
${\widetilde{a}}_{2}$	0	0	1	1	0	0
${\widetilde{a}}_{1}$	0	1	0	0	0	1
${\widetilde{a}}_{0}$	1	0	0	0	1	0

	No inversion			Inversion		
	0 (6)	4	2	5	3	1
${b}_{2}$	0	1	0	1	0	0
${b}_{1}$	0	0	1	0	1	0
${b}_{0}$	**0**	**0**	**0**	**1**	**1**	**1**

Significant values are in bold.

For comparison, in the same table, the values of the bits ${b}_{j}$ are indicated, which correspond to the values of the digital logarithms of the elements under consideration for the case $\theta =5$, i.e. the powers to which the primitive element $\theta =5$ must be raised in order to obtain the required element of the field $GF(7)$.

From Table 2 the value of the ${b}_{0}$ bit is determined only by the belonging of the considered field to one of the above sets. This corresponds to the factorization expressed by formulas (39) and (40). Therefore, the value of the bit ${b}_{0}$ corresponds to the signal generated at the output of block 2 of the circuit in Fig. 1.

In turn, this means that the operation of digital logarithm for the case under consideration is reduced to operations associated with setting the values of the two most significant digits at the output of the circuit in Fig. 1.

This problem is solved using switches 3_1,2_ as follows. Both among the input (for the switch block 3_1,2_), and among the output signals, there is a maximum of only one that differs from zero. Therefore, the transition from the set of logical variables ${\widetilde{a}}_{j}$ to the set of variables ${b}_{\mathrm{1,2}}$ can only be ensured by the nature of the connection between the elements of the circuit. This is shown in Table 3, which shows which output of the circuit in Fig. 1 must be supplied with that of the signals ${\widetilde{a}}_{j}$, which is non-zero for the digital logarithm operation to be performed.

**Table 3:** Correspondence between the numbers of outputs of the block elements 1_1–3_ and the numbers of inputs of the switch block 3_1,2_, corresponding to the digital logarithm operation.

	No inversion	Inversion
${\widetilde{a}}_{2}$	${b}_{1}$	${b}_{2}$
${\widetilde{a}}_{1}$	${b}_{2}$	–
${\widetilde{a}}_{0}$	–	${b}_{1}$

The signals from the outputs of elements 1_1–3_ are fed to switches 3_1,2_ which perform the following operation.

The first input of each of these switches receives the signal taken from the output of the element 1_1–3_ which corresponds to the first part of Table 3 (no inversion). The second input receives the signal corresponding to the second part of this table (inversion). The switch is controlled by a signal taken from block 2. If this signal equals zero, the state of the commutator output coincides with the state of the output of the first input, if one—with the state of the second input. As a result, the signals corresponding to the two high digits of value the digital logarithm in binary representation are formed at the outputs of the switches.

The lower digit is exactly equal to the value of logic variable formed at the output of element 2.

Thus, for the considered Galois field the execution of a digital logarithm calculation can be carried out by a quite simple scheme.

We emphasize that the presented scheme reflects a fundamental fact. The proposed approach to performing the digital logarithm operation is since the number $p-1$, where $p$ is prime, is decomposed into the product of some factors. Therefore, if the digital logarithm of the elements of an arbitrary Galois field is a mapping of the given field onto some ring, which, generally speaking, contains zero divisors and several idempotent elements.

The ring generated by the field $GF(7)$ in passing to the digital logarithm has two mutually canceling elements corresponding to simpler Galois fields. One of them corresponds to the field $GF(2)$. It is this fact that made it possible to classify the field elements (in the transition to digital logarithm) according to the value of the sum of the number of digits. A similar approach can be implemented for other Galois fields, since the digital logarithm in any case involves a mapping of its nonzero elements onto some algebraic ring.

Based on discussed above scheme, it is possible to propose various variants of other schemes, for example, implementing multiplication operation, six-digit logic operations, etc.

**Figure 2:**
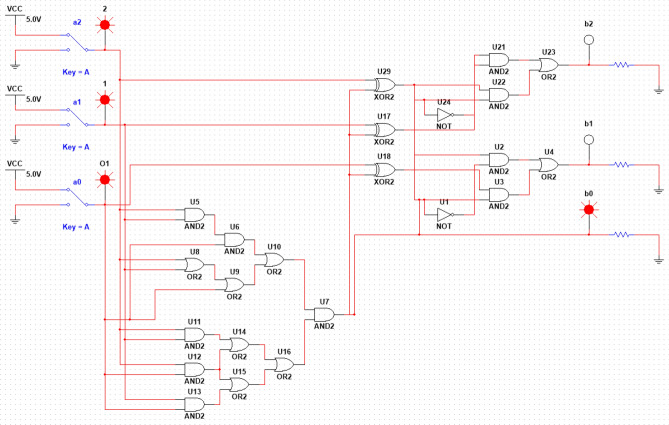
Digital logarithm performing scheme implemented by software NI Multisim.

Let's move on to the consideration of electronic circuits providing multiplication by modulo 6. This scheme is intended to show that it is possible to implement calculations according to formula (9) or similar ones even in the case when the number of arguments of a logical operation becomes sufficiently large. The circuit considered below corresponds to the representation of six-valued logic in (2.3) logic, which also corresponds to formula (38). Figure 2 shows a circuit that performs digital logarithm, implemented by software NI Multisim.

## Modulo 6 multiplication algorithm and multiplier circuit

The scheme considered below is intended to demonstrate the following fundamental circumstance, which is expressed by formula (26). It is possible to represent the element of the ring in the "hybrid" number system, formula (20). The digits of this number, if the operations proceed within the corresponding ring, can be handled independently.

This approach can be applied to various rings, but for the primary proof of its effectiveness in terms of implementation in the form of electronic circuits, it is permissible to restrict ourselves to the simplest example, which corresponds to the case of six-valued logic.

The modulo 6 multiplication algorithm involves the following operations, to which the individual blocks of the electronic circuit correspond. The list of operations includes:

- conversion of multiplied numbers in binary representation to binary numbers modulo 6,
- conversion of the numbers in binary encoding to encoding in (2;3)-logic representation,
- multiplication of numbers in (2;3)-logic representation,
- conversion of the number representation in the (2;3)-logic to the usual binary representation.

**Figure 3:**
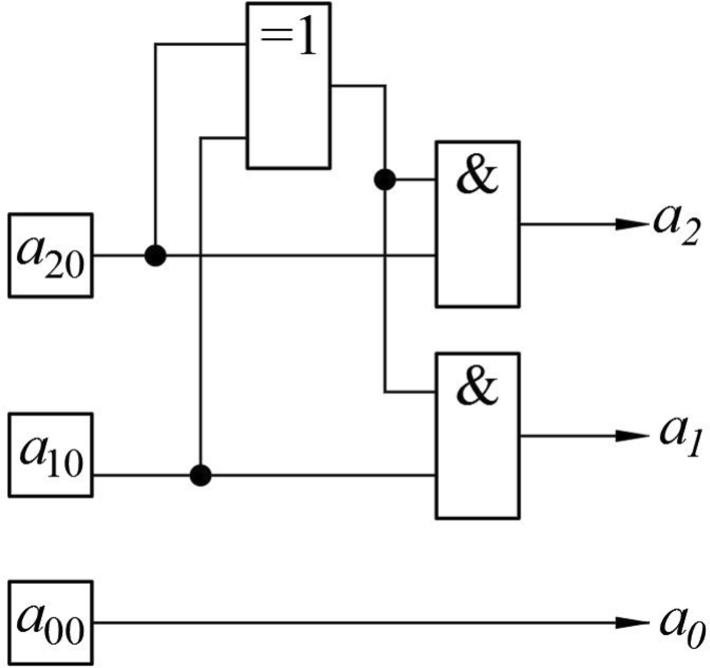
Schematic diagram of the block to convert the original numbers in binary representation to binary numbers modulo 6.

Scheme of the block to convert the initial numbers in the binary representation to binary numbers modulo 6 (Fig. 3) is since the numbers 6 and 7 in the binary representation are written as 110 and 111 respectively. When passing to calculations modulo 6, these numbers correspond to zero and one, i.e., for this case it is sufficient to replace the two most significant digits with zeros.

The EXCLUSIVE OR element in Fig. 3 compares the values of the two most significant digits. If they are the same, the output is a logical zero. The signal from the output of this element is fed to the elements of AND, the second output of which is fed signals corresponding to the values of ${a}_{01}$ and ${a}_{02}$, as a result of their output signals equal to ${a}_{01}$ and ${a}_{02}$, if the output of EXCLUSIVE OR is formed a logical unit (ie, the values of ${a}_{01}$ and ${a}_{02}$ do not coincide). On the contrary, if these values are the same, then at the output of the AND elements, logical zeros are formed.

Otherwise, the relationship between the values of variables on the input ${a}_{i0}$ and the output ${a}_{i}$ is given by the following logical formulas

44 $${a}_{2}={a}_{20}\left({a}_{10}+{a}_{20}\right),$$

45 $${a}_{1}={a}_{10}\left({a}_{10}+{a}_{20}\right),$$

46 $${a}_{0}={a}_{00}.$$

Let us consider an algorithm of functioning of the block of conversion of numbers in binary encoding to encoding in (2;3)-logic representation (Table 3). The first line of this table contains numbers n from 0 to 5; this set corresponds to operations modulo 6. The next three lines present binary variables corresponding to the bits when writing the number n in binary encoding.

The next two lines represent the numbers ${u}_{1}$ and ${u}_{2}$, corresponding to the bits modulo 2 and 3 in the (2;3)-logic representation, respectively. This corresponds to the representation of the considered numbers in the hybrid number system in accordance with formula (20). We emphasize that each of the digits in this formula corresponds to a certain Galois field, specifically, the six-valued logic leads to (2;3)-logic, which is formed by the Galois fields $GF(2)$ and $GF(3)$. Each of the numbers appearing in the bits of such a representation, in turn, can be represented in binary encoding. For the elements of the $GF(2)$ field, it is sufficient to use only one binary digit, the values of which are presented in the fifth row of this Table 4. The field $GF(3)$ needs two such bits.

**Table 4:** Transition from binary encoding to encoding in (2;3)-logic representation.

n	0	1	2	3	4	5
${a}_{2}$	0	0	0	0	1	1
${a}_{1}$	0	0	1	1	0	0
${a}_{0}$	0	1	0	1	0	1
${u}_{1}$	0	1	0	1	0	1
${u}_{2}$	0	1	2	0	1	2
${u}_{21}={b}_{2}$	0	0	1	0	0	1
${u}_{20}={b}_{1}$	0	1	0	0	1	0

Respectively, the two bottom lines represent the binary variables ${u}_{21}$ and ${u}_{20}$ corresponding to the representation of numbers ${u}_{2}$ in binary logic.

It follows directly from this table that in the (2;3)-logic representation ${u}_{1}={a}_{0}$

Let us consider next expression, where all quantities are treating as logical binary variables

47 $$q={a}_{2}+{a}_{1}{a}_{0}$$

Direct calculations show that this expression is equal to zero for numbers 0,1,2 and equal to one for numbers 3,4,5, which is proved by direct calculation. In particular, the value of ${a}_{2}$ (the most significant bit) is zero for numbers 0,1,2, and the product of ${a}_{1}{a}_{0}$ is also zero, since for numbers 0,1,2 only one of the two least significant bits is non-zero. The product ${a}_{1}{a}_{0}$ is equal to 1 only for the initial number 3 from the set under consideration, and for the initial numbers 4 and 5 it also vanishes. But, since the value ${a}_{2}$ also appears in the sum (47), then for the initial numbers 3,4 and 5, the $q$ parameter is equal to 1.

The $q$ parameter is auxiliary, its calculation allows one to find direct connection between values of digits ${b}_{1}$ and ${b}_{2}$ with initial values ${a}_{i}$.

Specifically, this relationship is expressed by the following logical formulas

48 $${b}_{2}={a}_{1}+q{a}_{0},$$

49 $${b}_{1}={a}_{0}+q,$$

50 $${b}_{0}={a}_{0}.$$

For the case of numbers 0, 1 and 2 $q=0$, and formulas (48) and (49) take the form that corresponds to the relationship between the considered quantities, determined by the second, third, and fourth columns of Table 4.

51 $${b}_{2}={a}_{1},$$

52 $${b}_{1}={a}_{0}.$$

Indeed, as Table 4 shows, for the numbers 0, 1 and 2, the value of the bit ${b}_{1}$ is the same as the value of ${a}_{0}$, and the value of ${b}_{2}$ is the same as the value of ${a}_{1}$.

For the case of numbers 3, 4 and 5 $q=1$, and formulas (51) and (52) take the form that corresponds to the relationship between the considered quantities, determined by the last three columns of Table 4.

53 $${b}_{2}={a}_{1}+{a}_{0},$$

54 $${b}_{1}={a}_{0}+1.$$

Indeed, as Table 4 shows, for the numbers 3, 4, and 5, the value of the bit ${b}_{1}$ is associated with the value of ${a}_{0}$ by a logical inversion (or sum modulo 2), and the value of ${b}_{2}$ can be obtained as the sum of the values of ${a}_{1}$ and ${a}_{0}$ modulo 2.

**Figure 4:**
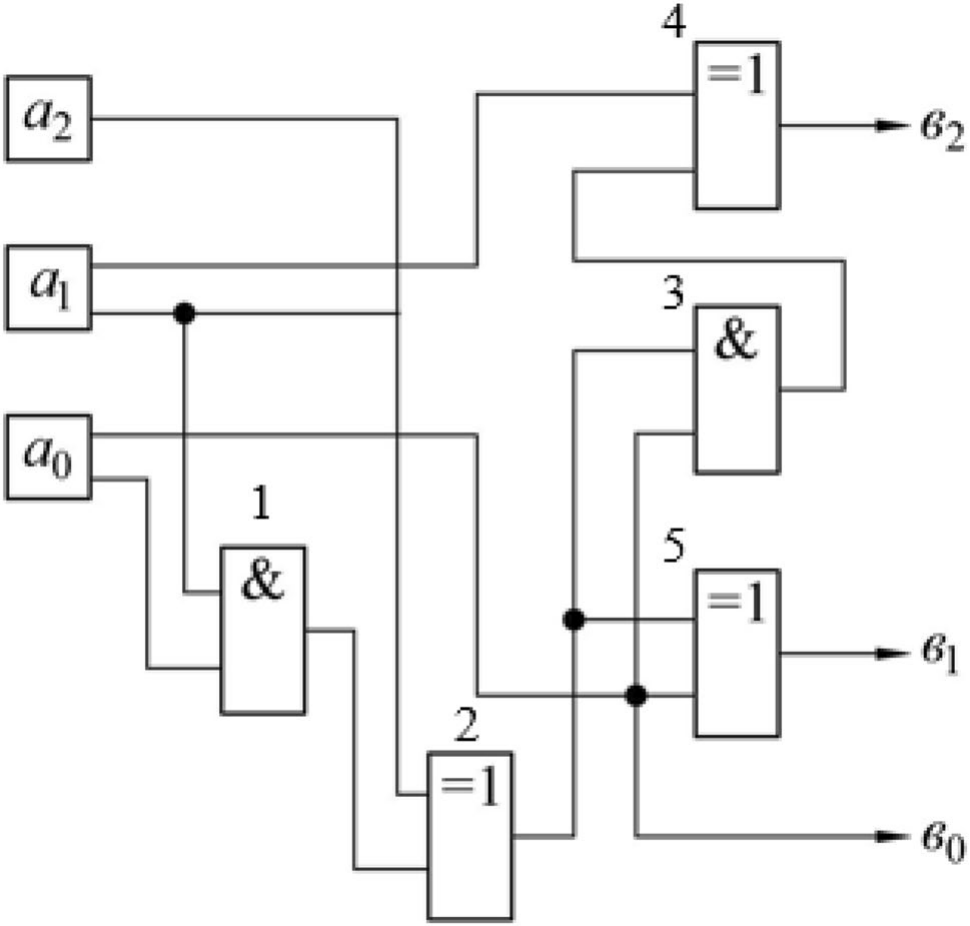
Schematic of the block to convert the numbers in binary encoding to encoding in the (2;3)-logic representation.

Calculations in accordance with formulas (48)–(50) are carried out by a fragment of the multiplier circuit modulo 6, shown in Fig. 4.

Due to relation (50), the lowest bit in the encoding used remains the same ${b}_{0}={a}_{0}$.

Two other binary digits corresponding to value ${u}_{2}$ are formed by next elements of the circuit, presented on Fig. 4.

The AND (1) and EXCLUSIVE OR (2) elements calculate the auxiliary boolean variable $q$. These elements perform the operation reflected by formula (47).

Signals ${a}_{0}$ and $q$ are sent to element AND (3), which corresponds to the calculation of the product $q{a}_{0}$ in formula (48). The signal from the output of this element is fed to the input of another EXCLUSIVE OR element (4) whose second input receives the signal corresponding to the value ${a}_{1}$, i.e., this part of the circuit implements the logical formula (48), i.e. at the output of the element, a signal is generated corresponding to the value ${b}_{2}$.

The signals ${a}_{0}$ and $q$ are also fed to the EXCLUSIVE OR element input (5), which thus performs the logical operation given by formula (49), i.e., its output generates a signal corresponding to the value of ${b}_{1}$.

The considered block (Fig. 4), in essence, performs only an intermediate transformation. Specifically, it provides a transition from the encoding corresponding to the variables of six-valued logic to the "hybrid" number system, reflected by formula (20), which in the case under consideration corresponds to the case of (2,3)-logic.

The main convenience of the transition to such logic is demonstrated by the modulo 6 multiplier circuit considered below, which is a circuit implementation of formula (25), which shows that when switching to a “hybrid” number system corresponding to the use of algebraic rings,

The main convenience of the transition to such logic is demonstrated by the modulo 6 multiplier circuit considered below, which is a circuit implementation of formula (25), which shows that when switching to a “hybrid” number system corresponding to the use of algebraic rings, operations are performed in terms of binary and ternary logic. The logic is reduced to a simpler one.

The resulting circuit of modulo 6 multiplier includes two similiar converters forming on its output one binary digit each corresponding to values ${u}_{1}^{(1)}$ and ${u}_{1}^{(2)}$, and two digits each corresponding to values ${u}_{2}^{(1)}$ and ${u}_{2}^{(2)}$. According to formula (15) presented above, the multiplication of these numbers is done independently, with one case multiplied modulo 2, and the other case multiplied modulo 3. To carry out the first of these operations it is sufficient to use the element AND (Fig. 4), i.e., ${z}_{0}={b}_{0}^{(1)}{b}_{0}^{(2)}$.

**Figure 5:**
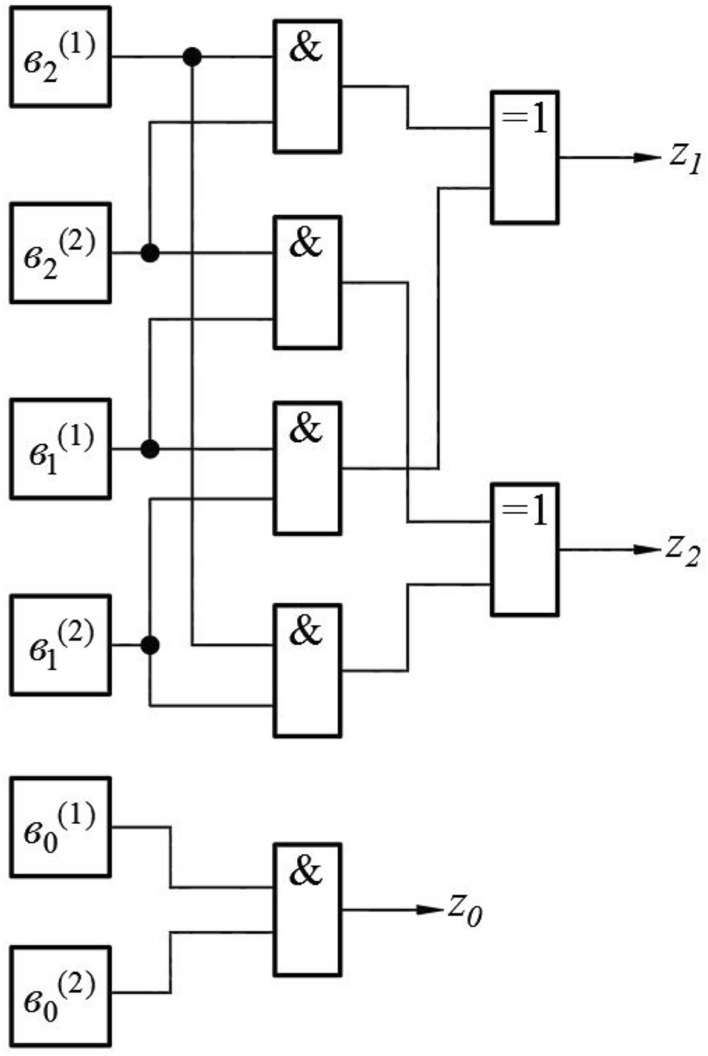
Diagram of the number multiplication block in (2;3)-logic representation.

Let's consider elements of the circuit that perform the second of these operations (Fig. 5). The table of multiplication by modulo 3 (Table 5) can be reduced to a form which specifies values ${z}_{2}$ and ${z}_{1}$, i.e., binary variables corresponding to high and low binary digits of multiplication result (Tables 6 and 7).

**Table 5:** Multiplication table modulo 3.

	0	1	2
0	0	0	0
1	0	1	2
2	0	2	1

**Table 6:** Table of least significant digits in modulo 3 multiplication.

	00	01	10
00	0	0	0
01	0	1	0
10	0	0	1

**Table 7:** Table of high digit values for multiplication by modulo 3.

	00	01	10
0	0	0	0
01	0	0	1
10	0	1	0

The above tables show that

55 $${z}_{1}={b}_{1}^{(1)}{b}_{1}^{(2)}+{b}_{2}^{(1)}{b}_{2}^{(2)},$$

56 $${z}_{2}={b}_{1}^{(1)}{b}_{2}^{(2)}+{b}_{2}^{(1)}{b}_{1}^{(2)},$$

where all values are treated as binary logic variables.

The scheme in Fig. 5 uses two AND elements for each of these operations (calculating the product modulo 2) and one OR circuit (calculating the sum).

Consider the inverse transformation to the representation of the result of the product in three-digit binary form (Fig. 4).

Possible encodings formed at the output of the circuit of Fig. 5 are shown in Table 8. The task is to convert them electronically into values corresponding to ${c}_{2}$ and ${c}_{1}$, which correspond to the product calculation result in the initial representation, ${c}_{0}={z}_{0}$ and require no transformation. The required result is shown in Table 9. The given values of logical variables, in essence, coincide with those reflected in Table 4, but we present them here for clarity of comparison again.

**Table 8:** Values of the logical variables corresponding to the integers at the output of the circuit in Fig. 5 (at the input of the circuit in Fig. 6).

$n$	0	1	2	3	4	5
${z}_{1}$	0	1	0	0	1	0
${z}_{2}$	0	0	1	0	0	1
${z}_{0}$	0	1	0	1	0	1

**Table 9:** The value of the logical variables that need to be generated at the output of the circuit of Fig. 6.

$n$	0	1	2	3	4	5
${c}_{2}$	0	0	0	0	1	1
${c}_{1}$	0	0	1	1	0	0

Directly from Table 8 you can see that the first and second triplets of numbers are classified by the values ${z}_{1}$ and ${z}_{0}$. If these values coincide, then the number in question takes the value 0, 1, or 2. If not—3, 4 or 5. Otherwise, the difference between one triplet and another is the value of the logical variable

57 $$w={z}_{1}+{z}_{0}.$$

For the first triplet $w$ is 0, and for the second triplet it is 1.

Further, for the first triplet, we can proceed from the pair ${z}_{1}$ and ${z}_{2}$ to the pair ${c}_{2}$ and ${c}_{1}$ by adding the value of ${z}_{1}$ as a logical variable to the value ${z}_{0}$. For the first triplet, the latter differs from zero only for $n=1$. That is, for the first three we have

58 $${c}_{2}={z}_{1}+{z}_{0},$$

59 $${c}_{1}={z}_{2}.$$

For the second triplet, the transition from the pair ${z}_{1}$ and ${z}_{2}$ to the pair ${c}_{2}$ and ${c}_{1}$ is provided by adding the value of ${z}_{0}$ to the two-digit binary number ${z}_{1}{z}_{2}$.

This corresponds to logical expressions

60 $${c}_{1}={z}_{2}+{z}_{0},$$

61 $${c}_{2}={z}_{1}+{z}_{0}{z}_{2}.$$

Using (57), the written formulas can be combined as

62 $${c}_{1}={z}_{2}+{\left({z}_{1}+{z}_{0}\right)z}_{0},$$

63 $${c}_{2}={z}_{1}+\left({z}_{1}+{z}_{0}+1\right){z}_{0}+\left({z}_{1}+{z}_{0}\right){z}_{0}{z}_{2}.$$

The last expression can be simplified

64 $${c}_{2}=\left({z}_{1}+{z}_{0}\right)\left(1+{z}_{0}\left(1+{z}_{2}\right)\right).$$

**Figure 6:**
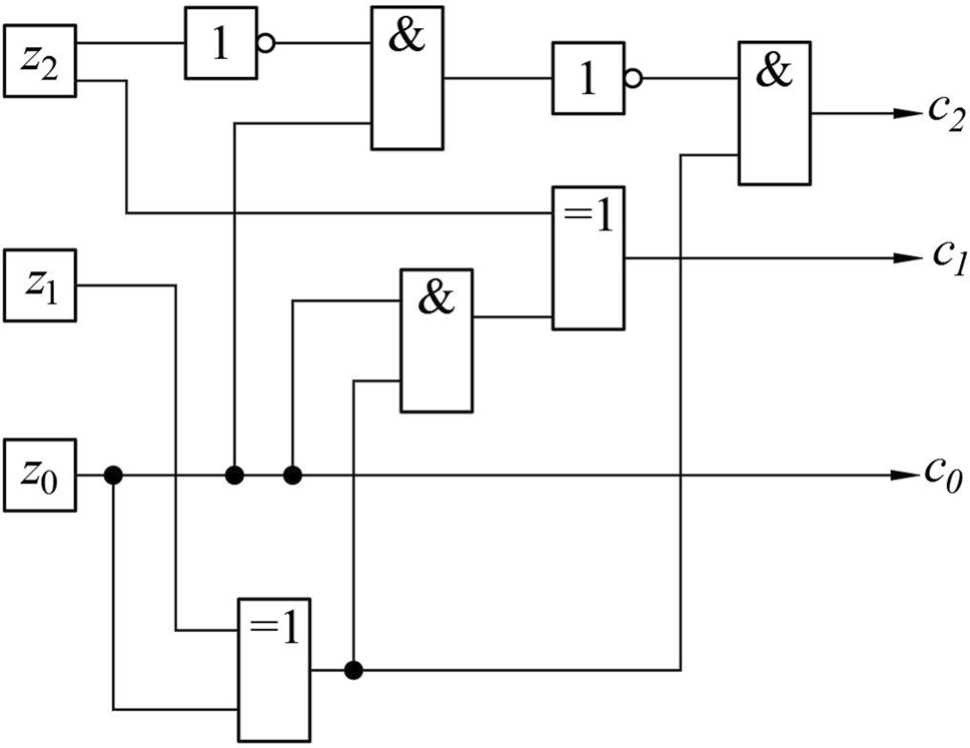
Schematic of the block for converting a number representation in (2;3)-logic to the usual binary representation.

These expressions are realized by the scheme shown in Fig. 6. It is taken into account that the logical addition with one corresponds to the inversion operation.

Thus, the operation of multiplication of two numbers modulo 6 can really be implemented by rather simple electronic circuits (including those implemented by simulation methods), which use only standard elements of binary logic.

**Figure 7:**
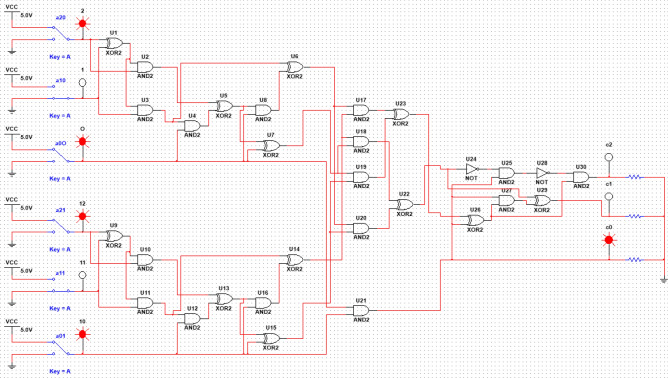
Modulo 6 multiplication scheme implemented by software NI Multisim.

The general electronic circuit that combines the blocks shown in Figs. 3, 4, 5, 6, shown in Fig. 7. This scheme is implemented using software NI Multisim, i.e., with its help, the efficiency of the proposed approach is proved directly.

## Conclusion

The increasing use of multivalued logics makes it relevant to reduce logical operations to algebraic ones. For those logics in which the number of values of a boolean variable is equal to ${p}^{n}$, where $p$ is a prime number and $n$ is an integer, this problem is solved by establishing a correspondence with Galois fields.

In this case, any logical operations can be reduced to operations of addition and multiplication in Galois fields, and there is an explicit tool that allows you to do this—the algebraic delta function, the usefulness and convenience of which was demonstrated in^20^. This function, in particular, allows one to write in explicit form an expression for an analogue of the Zhegalkin polynomial for arbitrary Galois fields.

However, there are several important logics (for example, six-valued and ten-valued logics), for which the reduction of logical operations to algebraic ones presents a certain problem.

This paper shows that it is possible to construct an algebraic delta function for the case when the number of values that a Boolean variable takes is $p-1$.

This case, among other things, demonstrates the fundamental difference between logics that allow direct comparison with Galois fields, and such logics, the number of variable values in which is not equal to ${p}^{n}$, where $p$ is a prime number and $n$ is an integer.

In the latter case, the reduction of logical operations to algebraic ones cannot be reduced only to addition and multiplication, it is required to use a larger number of operations. For $p-1$-logics such operations are the operation of digital logarithm and its inverse. Such operations, as shown in this work, allow us to bring the operations performed for $p-1$-logic to the operations performed in the field $GF(p)$.

The adequacy of the proposed approach is demonstrated on the example of electronic circuits, which allow reducing the operations of six-valued logic to algebraic ones, which can be technically implemented even based on standard logical electronic components.

The proposed approach is of interest for the development of logic circuits based on a non-standard element base, for example, on a quasi-biological basis. The development of such systems returns to the question of how exactly computer systems should be implemented that are complementary to the usual decimal number system. There is no need to prove that bringing calculations in the binary number system to decimal form requires additional computer resources. New computing systems may well be based on a combined number system, on (2.5), a logic that is complementary to the Galois field $GF(11)$.

The advantage of this approach is the simplification of addition and multiplication operations by technical devices, which follows from formulas (25), (26), as well as the possibility of its extension to other logics, the number of variable values in which is equal to the product of prime numbers ${q}_{1}{q}_{2}\dots {q}_{s}$, the value of which itself equals $p-1$, where p is a prime number: ${q}_{1}{q}_{2}\dots {q}_{s}=p-1$.

Examples of such numbers are presented in Table 10.

**Table 10:** Factors ${q}_{i}$ giving examples of numbers of the form ${q}_{1}{q}_{2}\dots {q}_{s}=p-1$.

${q}_{4}$	${q}_{3}$	${q}_{2}$	${q}_{1}$	$M={q}_{1}{q}_{2}\dots {q}_{s}$	$p$	$Mp$
–	–	3	2	6	7	42
–	–	5	2	10	11	110
–	–	11	2	22	23	506
–	5	3	2	30	31	930
–	7	3	2	42	43	1806
–	11	3	2	66	67	4422
–	7	5	2	70	71	4970
–	17	3	2	102	103	10,506
–	13	5	2	130	131	17,030

Indeed, in this case it becomes possible to reduce the operations of logic, the number of values of the variables of which is equal to $p-1$, to operations in algebraic rings corresponding to the product ${p}_{1}{p}_{2}\dots {p}_{s}$.

This significantly expands the list of multivalued logics, the operations in which are reducible to algebraic ones, which, however, requires additional research.

In addition, the use of multi-valued logics is a promising tool for improving the means of digital signal and image processing. Solving problems of this kind requires reducing logical operations to algebraic ones, which determines the prospects for further research in this direction. In particular, the task of further weakening the restrictions imposed on the number of values that a boolean variable can take is topical.

## Data Availability

All data generated or analysed during this study are included in this published article.
